# Development and Validation of a LC-MS/MS Technique for the Analysis of Short Chain Fatty Acids in Tissues and Biological Fluids without Derivatisation Using Isotope Labelled Internal Standards

**DOI:** 10.3390/molecules26216444

**Published:** 2021-10-26

**Authors:** Shikha Saha, Priscilla Day-Walsh, Emad Shehata, Paul Anthony Kroon

**Affiliations:** 1 Quadram Institute Bioscience, Norwich Research Park, Norwich NR4 7UQ, UK; shikha.saha@quadram.ac.uk (S.S.); priscilla.day-walsh@quadram.ac.uk (P.D.-W.); emad.shehata@quadram.ac.uk (E.S.); 2 National Research Centre, Chemistry of Flavour and Aroma Department, Dokki, Cairo 12622, Egypt

**Keywords:** gut microbiota, kidney, diabetes, neurodegenerative, cardiovascular, plasma, milk, lactate, butyrate, acetate, propionate

## Abstract

The gut microbiota is critical to the maintenance of physiological homeostasis and as such is implicated in a range of diseases such as colon cancer, ulcerative colitis, diabetes, cardiovascular diseases, and neurodegenerative diseases. Short chain fatty acids (SCFAs) are key metabolites produced by the gut microbiota from the fermentation of dietary fibre. Here we present a novel, sensitive, and direct LC-MS/MS technique using isotopically labelled internal standards without derivatisation for the analysis of SCFAs in different biological matrices. The technique has significant advantages over the current widely used techniques based on sample derivatization and GC-MS analysis, including fast and simple sample preparation and short LC runtime (10 min). The technique is specific and sensitive for the quantification of acetate, butyrate, isobutyrate, isovalerate, lactate, propionate and valerate. The limits of detection were all 0.001 mM except for acetate which was 0.003 mM. The calibration curves for all the analytes were linear with correlation coefficients r^2^ > 0.998. The intra- and inter-day precisions in three levels of known concentrations were <12% and <20%, respectively. The quantification accuracy ranged from 92% to 120%. The technique reported here offers a valuable analytical tool for use in studies of SCFA production in the gut and their distribution to host tissues.

## 1. Introduction

The gut microbiota has emerged as critical to human metabolism and physiological homeostasis and is thus implicated in a range of metabolic, inflammatory, and neurological diseases [1,2,3]. Short chain fatty acids (SCFAs), which include butyrate, propionate and acetate, are key metabolites produced by the gut microbiota from the fermentation of dietary fibre and resistant starch [4,5]. While not necessarily a SCFA, lactate produced from dietary fibre can also serve as a precursor for SCFAs and as a marker of lactic acid bacteria such as Lactobacillus and Lactococcus among many others [6]. Additionally, microbial fermentation of dietary amino acids such as leucine, isoleucine and valine result in the formation of branched short chain fatty acids (BSCFAs) as stereo isomers of the SCFAs butyrate and valerate, which have been proposed as markers of microbial protein metabolism with a particular emphasis on their positive correlation with obesity, ageing, and metabolic diseases [7,8].

As well as providing energy for colonic cells important for gut barrier integrity, SCFAs have emerged as energy substrates for colonic and liver cells and as signalling molecules influencing a range of metabolic and physiological pathways in the liver, brain, kidney, and the immune system [3,9]. Additionally, due to their low pH, SCFA are critical in preventing the colonization of pathogenic microbes [10]. In vitro and in vivo studies have shown that the disruption of microbial composition and diversity can affect the bioavailability of both SCFA and BSCFAs leading to diseases such as colon cancer, ulcerative colitis, diabetes, cardiovascular diseases, and neurodegenerative diseases [3,11]. While the bioavailability and function of SCFA and BSCFA is usually extrapolated from caecal contents and plasma levels, little is known regarding the bioavailability of these compounds in peripheral organs such as the liver, kidney, and the brain, possibly due to methodological limitations. Nevertheless, SCFA have been proposed to elicit their physiological effects by interacting with G protein coupled receptors (GPR) such as GPR41 and GPR43 and serotonergic receptors [12]. Therefore, it is important to know physiological levels of SCFAs, lactate and BSCFA (referred to as SCFAs) in these tissues as this may help to determine whether their effects are local, directly inducing cell signalling at the target tissue or are mediated remotely through second messenger signalling molecules.

Several techniques have been used for the analysis of SCFA in biological fluids including nuclear magnetic resonance (NMR) spectroscopy, capillary electrophoresis (CE), gas chromatography mass spectrometry (GC-MS), high performance liquid chromatography (HPLC), size-exclusion chromatography and liquid chromatography-tandem mass spectrometry (LC-MS/MS). The drawbacks of utilising the first four techniques were critically reviewed by Primec, et al. [13]. While to date GC-MS remains an instrument of choice for SCFA analysis owing to its affordability, high sensitivity and resolution, the laborious multi-step sample cleanup process which typically involves ultra-sonication, shaking during incubation, centrifugation followed by filtration, derivatisation, drying and sample dilution may lead to poor analyte recovery and reductions in reproducibility and accuracy, as well as low throughput. This makes this technique far from ideal for the analysis of these metabolites in a large number of samples [13,14,15]. Likewise, HPLC has been used for the analysis of SCFAs in complex biological samples with clean-up steps and drawbacks similar to those used in GC-MS [16,17,18]. While a number of studies have also employed LC-MS/MS, the analysis of SCFA has still required sample filtration and derivatisation using one of the available reagents such as tris (2,4,6-trimethoxyphenyl) phosphonium propylamine (TMPP) bromide or carboxylic acids such as 4-[2-(N,Ndiethylamino)ethylaminosulfonyl]-7-(2-aminoethylamino)-2,1,3-benzoxadiazole (DAABD-AE) as well as 3-nitrophenylhydrazone (3NPH) followed by the addition of 3-nitrophenylhydrazine hydrochloride *N*-(3-dimethylaminopropyl)-*N*′-ethylcarbodiimide hydrochloride [19,20,21,22,23]. The only LC-MS/MS technique that has been utilised to measure SCFA in plasma, without derivatisation, required a post neutralization technique before MS detection and with the use of hydrochloric acid (HCl) in the mobile phases, which is a very harsh condition for HPLC columns and would shorten its lifespan should the use of large sample numbers be required [24]. The technique monitored only ammonium adducts of SCFAs with only propionate, acetate, butyrate and valerate being detected. Additionally, most of the other aforementioned techniques did not simultaneously measure lactate and BSCFAs. Simultaneous detection of SCFA, lactate and BSCFAs is also important as several studies have shown that while the SCFA may not change in response to treatment, BSCFAs may change, and vice versa [23,25,26]. Thus, it is evident that there is a requirement for a robust, sensitive, high throughput, column friendly technique for measuring not only SCFAs but also lactate and BSCFAs in biological samples including plasma, faeces, in vitro fermented faecal samples, milk, and other tissues such as liver, kidney, skeletal muscle, etc.

Here we present a sensitive, simple, and high throughput technique without derivatisation for LC-MS/MS (MRM) based analysis of SCFAs, in mouse and human faecal and mouse liver, kidney, brain, skeletal muscle, spleen samples and microbial fermentation media.

## 2. Results

### 2.1. Mass Spectrometry Conditions

To obtain precursor and products ions of acetate, lactate, propionate, butyrate, isobutyrate, valerate and isovalerate in electrospray ionization mode, Agilent MassHunter automated Optimizer software was used. The collision energy was used from 0 to 80 by 10 CE step increments in negative and positive polarity modes. The fragmentor value was constant at 380 V. The positive polarity produced more intense product ions for all compounds except lactate. Lactate produced a more intense peak in negative ion mode. The precursor ion and the product ion with the highest signal to noise (S/N) value and the highest peak intensity was selected for the quantifier ion and the other product ion was selected for the qualifier ions. Table 1 summarizes the monitored ions and the optimized MS operating parameters of the analytes and internal standards.

### 2.2. Chromatographic Separations

We aimed to develop a technique with a short run time and good sensitivity for the analysis of SCFAs, stereo isomers and lactate in a wide range of matrices. As such the Waters Acquity UPLC HSS T3 C18 1.8 µm, Acquity UPLC BEH C18 1.7 µm, Kinetex-C18 1.7 µm, Kinetex-PFP 1.7 µm, Kinetex-XB-C18 1.7 µm, Luna Omega 1.6 polar C18 and Thermo Scientific Hypercarb (Porous Graphatic Carbon, PGC) columns were tested to achieve an optimal retention of SCFAs. Good retention and peak shapes were achieved on Kinetex-PFP 1.7 µm, Kinetex-XB-C18 1.7 µm, and Luna Omega 1.6 polar C18 column using 0.1% formic acid in both water and acetonitrile. However, butyrate and isobutyrate, valerate and isovalerate isomeric compounds could not be separated by these columns. Therefore, another column was tried to separate stereo-isomeric compounds. Thermo Scientific Hypercar (porous graphitic carbon, PGC) 3 µm (50 mm × 2.1 mm) column and guard column was used to separate these seven analytes. Using this column, a good separation, good retention time and peak shape were obtained for all analytes. Isomeric compounds also were separated using mobile phase 0.1% formic acid in water and acetonitrile; Hypercarb column surface is a flat sheet of hexagonally arranged carbon atoms with a very large polynuclear aromatic compounds which is stereo-selective and allowed separation of geometric-isomers [27]. Additionally, it is stable at all pH ranges (0–14), high temperatures and aggressive mobile phases.

**Figure 1:**
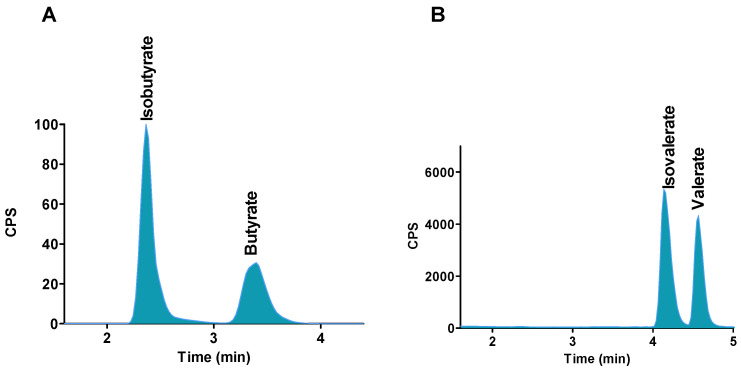
Separation of isomers and stereoisomers of SCFAs using PGC column. (**A**) Isobutyrate and butyrate, (**B**) Isovalerate and valerate.

The separation of isobutyrate and butyrate, isovalerate and valerate without derivatisation on PGC column is shown in the Figure 1A,B. Chromatographic separation for these isomers is necessary for accurate quantification because isobutyrate and butyrate molecular ions and fragmentations are the same and isovalerate and valerate have the same parent ions and fragmentation ions. Isomeric compounds could not be separated using normal silica base C18 column without derivatisation of SCFA.

### 2.3. Method Performance

Linearity, accuracy, precision, recovery and sample stability were studied for validation. These are acceptable criteria for validation of developed methods for publication and future use in biological samples [28]. Method validation was performed using human batch fermentation (colon model) media, mouse faecal samples and various mouse tissues by choosing an appropriate matrix [29].

#### 2.3.1. Linearity and Sensitivity

Endogenous SCFAs are presented in all biological matrices, calibration curves were constructed in aqueous solutions using a stable isotopically labelled internal standard technique for quantification. Isotopically labelled internal standards and analytes contributed to similar chromatographic properties and mass spectroscopic responses, which allowed for the correction of matrix effect variation between the different matrices and the aqueous calibration curve [30,31]. A wide range of concentrations (0.001 mM–10 mM) were studied for calibration curves for all compounds. The least-squares regression calibration curve was r^2^ = 0.998 for all compounds (Table 2).

The optimization software for MRM transition optimization for acetate, butyrate, isobutyrate, isovalerate, lactate, propionate and valerate showed the specific and most sensitive transition at *m/z* 61 > 43, 89 > 43, 89 > 43, 103 > 43, 89 > 43, 75 > 29 and 103 > 75 respectively. All mentioned SCFAs were well retained and separated well on the PGC column using a gradient mobile phase with the overall runtime of 10 min. LOD and LOQ values for all compounds are shown in the validation data (Table 2).

#### 2.3.2. Precision and Accuracy

To evaluate intra-day precision replicate (*n* = 6), analysis of three levels of known concentrations (low, medium and high) were spiked in 0.5% orthophosphoric acidified water and analysed by the current techniques. The precision was calculated from the relative standard deviation. The CV (%) was less than 12% for intra-day precision. Inter-day precision was evaluated by analysing the same three levels of concentration samples in acidified water for 5 days. The CV (%) values were <20%. Precision and accuracy data are presented in Table 2.

#### 2.3.3. Carry-Over Effect

For the quantification of metabolites in biological samples by LC-MS/MS, the carry over effect is a common problem. An agilent 1290 series high performance auto sampler with an injection program was used to minimize carry-over effects in this study. No signals were detected in any of the blank samples run amongst SCFA-containing samples, indicating that there was little or no carry over occurring.

#### 2.3.4. Recovery and Matrix Effect

For recovery calculation, known concentrations of individual SCFAs were spiked into the appropriate matrices, and after extraction the samples were quantified using an LC-MS/MS techniques. Nine different matrices were used to determine recovery. The lowest recovery was observed with lactate in the mouse brain samples at about 47%. (See Table 3 and Table 4).

The matrix effect on individual analytes were assessed to compare the peak area of the respective isotopically labelled internal standard in post extracted matrix to that in aqueous solution. No matrix effect was noticed for isobutyrate and propionate analysis in Table 3 matrices. However, in the colon model fermented sample, very little matrix effect was found for these compounds, and no matrix effect was observed for butyrate in this matrix (Table 4). The highest matrix effect was observed in spleen matrix for butyrate (76% signal response compared to water), isovalerate (80%) and valerate (84%) analysis, (see Table 3 and Table 4).

#### 2.3.5. Sample Stability

All standards and isotope labelled internal standards were prepared in water except for isobutyrate, valerate and isovalerate and their internal standards were prepared in ethanol. Density and purity were considered for stock solution preparation. Stock solutions were kept at −20 °C for 6 months, and no changes were observed. The stability of the extracted samples was evaluated at 4 °C for 72 h. No significant changes were noticed in sample stability over this time period.

### 2.4. Quantification of SCFAs in Human Colon Model Fermentation Samples

**Figure 2:**
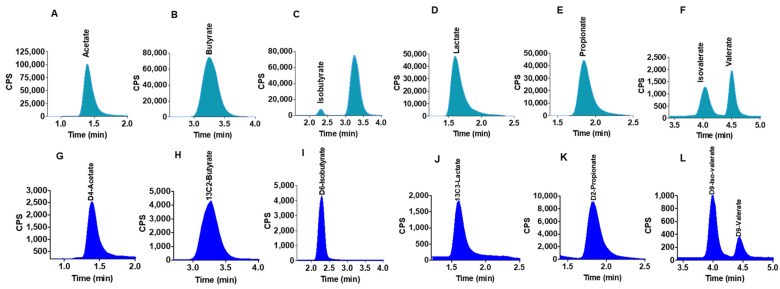
LC-MS generated SCFA peaks in colon model fermentation samples. (**A**–**F**) peaks detected in colon model fermentation samples. (**G**–**L**) corresponding labelled internal standards of SCFA in colon model samples.

Colon model fermentation media from three donors each were carried out in duplicates at different time points (0 to 48 h), samples (*n* = 6) were diluted with the addition of 0.5% orthophosphoric acid in methanol containing all isotopically labelled internal standards and subsequently analysed on the LC-MS/MS. All SCFAS peaks were identified in all samples. Figure 2A–F show the 7 SCFA peaks and retention times for all analytes detected in a colon model sample. Figure 2A–L show the corresponding labelled internal standards of SCFAs in colon model samples. The calibration curves constructed from the authentic standards with concentrations of 0–10 mM were linear with a correlation r^2^ value of >0.999.

### 2.5. Quantification of SCFAs in Mouse Liver Samples

**Figure 3:**
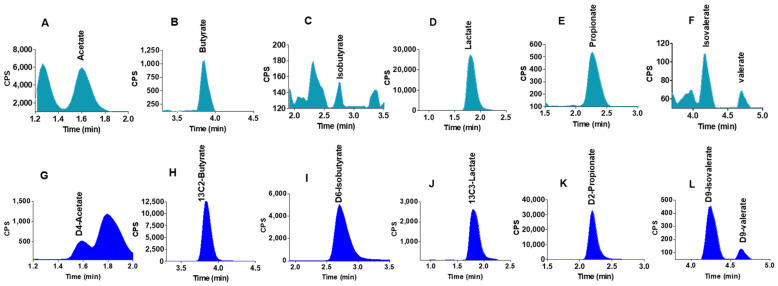
LC-MS generated SCFA peaks in mouse liver samples. (**A**–**F**) peaks detected in mouse liver samples. (**G**–**L**) corresponding labelled internal standards of SCFA in mouse liver samples.

Liver samples (*n* = 15) from animal study were extracted by 0.5% orthophosphoric acid and analysed by the current LC-MS/MS method. All analytes were detected in the liver samples. Figure 3A–F show the detected analytes in the liver samples and Figure 3G–L show the corresponding isotopically labelled internal standards in the liver samples.

## 3. Discussion

The LC-MS/MS technique has great advantages in providing analytical capacity for the simultaneous detection of human metabolites in different biological matrices [32]. SCFAs have been previously analysed in different materials by HPLC using derivatisation techniques which are less sensitive due to the requirement of UV detection and laborious sample preparation as well as long run times (~65 min) rendering them low throughput. Although GC-MS has been widely employed to analyse SCFAs, it has a wide range of drawbacks as described by Primec, et al., 2017 [13]. Since then another technique utilising GC-MS without derivatisation was developed but still required liquid-liquid extraction [33]. Further, LC-MS techniques requiring sample derivatisation have also been used, some involving a very short run time (14 min) and covering a wide range of gut derived metabolites, but once again they require longer sample preparation including sample filtration, which is time consuming and laborious [21,34,35,36]. A technique with a non-derivatisation step has been developed to analyse SCFAs in plasma using LC-MS, but this requires post neutralization techniques before MS detection and with the use of HCl in the mobile phases, which is very harsh for the HPLC column [24]. Additionally, this technique did not involve simultaneous analysis of stereo isomers and lactate in a wide range of matrices. Studies requiring the analysis of a large number of samples in a wide range of matrices would need the implementation of a fast and simple sample preparation and non-derivatisation high-throughput technique that prevents sample loss. The LC-MS techniques reported to date for quantifying SCFAs have not been tested or validated for use with a wide range of tissues and complex fermentation media which is paramount to future studies that aim to understand the physiological role of these microbial derived metabolites in health and disease. Here we provide a technique with a short run time and good sensitivity for the analysis of SCFAs, stereo isomers and lactate in a wide range of matrices. A brief summary of the aforementioned techniques in comparison to the current one is shown in Table 5.

Thus, we have developed a new validated technique which does not require sample derivatisation and allows the analysis SCFAs and related metabolites simultaneously in different matrices, employing isotopically labelled internal standards to correct for error in sample preparation, matrix effect and instrumental variation using PGC column. Researchers can also use the common C18-phase columns (Kinetex-XB and Luna Omega polar) or Kinetex-PFP column for the analysis of the five compounds; acetate, butyrate, lactate, propionate and valerate without isomeric compound (isobutyrate and isovalerate) analysis in different matrices, although this would not allow the separation of the stereoisomers.

To our knowledge, this is the first report describing a LC-MS/MS technique for the analysis of SCFAs and related metabolites with a wider application in complex biological fluids and tissues. This technique requires less laborious, fast sample preparative steps with a short LC-MS/MS run time (T = 10 min), allowing the analysis of a large number of samples from a wide range of tissues and fluids within a day. The LC-MS/MS technique described in this study allows the analysis of very low levels of SCFAs (0.001 mM) in different matrices. To assess matrix effects, the same concentration of corresponding isotopically labelled internal standards were spiked in different matrices after carrying out extraction as described in the methods section. We found a significant difference in the peak area of labelled internal standards in the different matrices using the electrospray ionization source. Therefore, isotopically labelled internal standards were used, as they are very important for accurate quantification of these compounds in different matrices. While a few studies have demonstrated the bioavailability of SCFA in the human brain and in portal, hepatic, and venous blood, to our knowledge this is the first study to show direct bioavailability of SCFAs in other mouse tissues including the liver, skeletal muscle, kidney and spleen [37,38,39].

**Methodology Limitations**: It is noteworthy that some areas of method performance are not ideal, for example the matrix effect in the brain, liver and plasma samples may lead to a low recovery rate for lactate. However, this should not compromise the accuracy for lactate measurements in these tissue samples, as when analyses are carried out in the same matrix, the low recovery rate does not affect quantification accuracy, particularly as this effect is counteracted by the use of isotopically labelled internal standards with quantification carried out using a standard curve. Nevertheless, this stresses the requirement that standard curves be carried out in the same matrices. The poor recovery of lactate, which was measured simultaneously with SCFAs, is consistent with a previous study where lactate recovery from supernatants of bacterial culture was 25% [40]. In the CG-MS techniques described by Primec, et al. although lactic acid was measured from the same samples as SCFA, it is important to note that lactate was measured separately after methylation. Low recovery has no hinderance in measuring the analyte as long as it is consistent across the batches [13]. To our knowledge, no other techniques employing LC/MS/MS have analysed lactate along with SCFAs, although lactate has previously been analysed using LC/MS/MS along with other organic acids [32]. Future studies may wish to examine the recovery of lactate after pretreatment using trichloro acatic acid, formic acid or perchloric acid.

## 4. Materials and Methods

### 4.1. Chemical and Reagents

Acetic, butyric, *d*_4_-acetic, ^13^C_2_-butyric, isobutyric, isovaleric, lactic, propionic, and valeric acids, were purchased from Sigma^®^ (Dorset, UK). D_6_-isobutyric, D_9_-isovaleric,^13^C_3_-lactic, D_2_-propionic and D_9_-valeric acids were purchased from Toronto research chemicals (Toronto, Canada). Ortho-phosphoric and formic acid was obtained from Lichropur (Dorset, UK). Semi-skimmed milk was bought from ALDI supermarket (Essen, Germany). Human plasma (K2EDTA) was purchased from BIoIVT (Royston, UK). All solvents with high purity grade were used for LC-MS/MS analysis.

### 4.2. Colon Model Fermentation Media and Mouse Tissue Samples

#### 4.2.1. Colon Model Study Participants

An in vitro batch fermentation (human colon) model as described by Day-Walsh et-al, 2021 was used to study microbial production of SCFAs, lactate and BSCFAs without supplementation of complex carbohydrates [41]. Fresh faecal samples were obtained from participants who were recruited onto the QIB Colon Model study. The study consisted of men and women aged 18 years or older who met the following inclusion criteria: a normal bowel habit with an average Bristol Stool Chart type of 3–5, they had regular defecation of between three times per day and three times per week and had no diagnosed chronic gastrointestinal health problems such as inflammatory bowel disease, irritable bowel syndrome, or celiac disease. Prior to sample donation, participants were not pregnant, or breast feeding, had not taken antibiotics or probiotics within the four weeks and had no gastrointestinal complaints such as vomiting or diarrhea within the last 72 h, and had not recently had an operation requiring general anaesthetic. Samples were collected after an informed consent from all participating subjects and a trial approval (registered at http://www.clinicaltrials.gov (accessed on 21 October 2021) (NCT02653001). Fresh human faecal slurry from three different donors were used for the study in duplicates. Samples were collected at 0, 4, 8, 12, 24 and 48 h and kept at −20 °C immediately until analysis.

#### 4.2.2. Animals and Sample Processing

All experimental protocols and procedures were reviewed and approved by the Animal Welfare and Ethical Review Body (AWERB) at the University of East Anglia, and they were conducted in accordance with the provisions of the Animals (Scientific Procedures) Act 1986 (ASPA) and the LASA Guiding Principles for Preparing for and Undertaking Aseptic Surgery (2010) as described by Day, et al., 2018 [42]. Tissues were collected and frozen on dry ice immediately and transferred to −20 °C until processing. Faecal samples were collected prior to animal sacrifice.

#### 4.2.3. Sample Processing

SCFAs from all samples were extracted in 0.5% orthophosphoric acid as outlined by Zhao et al., 2005 and García-Villalba et al., 2012 [15,43]. Samples were thawed on ice, centrifuged and 10 µL of the supernatant was mixed with 90 µL 0.5% orthophosphoric acid containing all isotopically labelled internal standards (5 mM for acetate, 0.25 mM for lactate and 0.5 mM the other five). Samples were further centrifuged at 15,000 rpm for 10 min and the supernatant was transferred to the chromatography vials for analysis using LC-MS. For tissue samples, tissues were pulverized using a pestle and mortar under dry ice and mixed into a homogenous powder. 30 mg of each tissue was mixed with 200 µL of 0.5% orthophosphoric acid in water and homogenized using the Precellys 24 lysis homogeniser at 6000 rpm for 2 cycles for 30 s (Bertin Technologies, Montigny-le-Bretonneux, France). After centrifugation for 10 min at 4 °C, 45 µL of the supernatant was mixed with 5 µL of 0.5% orthophosphoric acid containing all isotopically labelled internal standards (conc. 0.5 mM for all except lactate, lactate conc. was 0.25 mM). Other sample extraction methods including 100% methanol in 0.5% orthophosphoric acid (85% orthophosphoric acid diluted in methanol) and 50% methanol in 0.5% orthophosphoric acid were also trialed, but these gave a cloudy mixture that did not separate completely even after centrifugation at 15,000 rpm for 15 min.

Plasma and semi-skimmed milk (50 µL) were mixed with 100 µL methanol containing isotopically labeled internal standards (1 mM for acetate, 0.25 mM lactate and 0.5 mM other analyses) and vortexed. Samples were kept for 5 min on the ice to complete protein precipitation. After centrifugation for 10 min at 4 °C at 15,000 rpm, samples were transferred to HPLC vials and analysed by the present LC-MS/MS technique.

### 4.3. LC-MS/MS Analysis

All SCFAs authentic standards were reconstituted in acidified Milli-Q^®^ water to prepare stock solutions at the concentration of 100 mM. All stock solutions were kept at −20 °C. A standard curve was produced from stock solutions daily. A standard curve was produced with serial dilutions from the highest concentration (10 mM to 0.001 mM). All serial dilutions were prepared prior to each run. An Agilent 6490 Triple Quad MS mass spectrometer (Agilent Technologies, Santa Clara, CA, USA) equipped with an Agilent 1290 HPLC system (Agilent Technologies, Santa Clara, CA, USA) was used for the analysis of SCFA. The LC flow rate was 0.15 mL/min. The column used for the analysis was a Thermofisher PGC 3 µm (50 mm × 2.1mm) or Phenomenex 1.8 µm (100 mm × 2.1 mm PFP column with guard column. The column temperature and auto sampler were maintained at 40 °C and 4 °C, respectively. 2 µL was used for the injection volume. Samples were analysed using 0.1% formic acid in water (mobile phase A) and 0.1% formic acid in acetonitrile (mobile phase B). The gradient was started with 0% B, increased 60% B within 4 min, after washing for 2 min using 100% mobile phase B and equilibration was for another 4 min using 100% mobile phase A. The equilibration time was kept a little bit longer (4 min), because of PGC column packing material. The total run was 10 min. The 6490 MS/MS system was equipped with an electrospray ionization (ESI) source operated in positive and negative-ion detection mode. Nitrogen gas was used for nebulation, desolvation, and collision. The analytes were monitored in multiple-reaction monitoring (MRM) mode. The MRM precursor, product ions and collision energy were optimized by Agilent optimizer software. The transitions of precursor ions to product ions (*m/z*) and some optimized MS operating parameters of the analyte are described in Table 1. The source parameters were: gas temperature of 200 °C with a gas flow of 16 L/minute, a sheath gas temperature of 300 °C with a sheath gas flow of 11 L/minute, a nebuliser pressure of 50 psi and capillary voltage of 3500 V for positive polarity, and a Nozzle Voltage 1000 V. The iFunnel parameters were: high pressure radio frequency (RF) of 150 V and low-pressure RF of 60 V. The LC eluent flow was sprayed into the mass spectrometer interface without splitting. Identification was achieved based on retention time of authentic SCFA standards and by product ions monitor.

### 4.4. Method Validation

#### 4.4.1. Linearity

Seven authentic standards were spiked in acidified water to construct calibration curves for all compound analysis. The concentrations versus peak area ratio (analyte peak area/internal standard peak area) were plotted to construct the calibration curves.

#### 4.4.2. Sensitivity

Diluted solution of individual analytes was injected to get LOD and LOQ values. LOD was calculated as signal to noise ratio at least three times higher than the baseline noise. LOQ was calculated at a signal to noise ratio 10 times higher than the baseline noise of each compound.

#### 4.4.3. Precision and Accuracy

Intra-day precision and accuracy were calculated by analysis of replicates spiked in acidified water at concentrations of 0.01 (L), 0.5 (M) and 5 (H) mM for all SCFA (*n* = 6 at each level) on the same day. To assess the inter-day precision and accuracy, spiked replicates of the same concentration level (*n* = 6) were analysed on five different days. The relative standard deviation (R.S.D. %) of the replicate analyses was used for precision calculation. Accuracy was calculated by comparison of expected concentrations with the measured concentrations of the spiked samples. A R.S.D. % of 20% was deemed acceptable for both precision and accuracy.

#### 4.4.4. Carry-Over Effect

To assess carry-over effects, water was injected after an injection of the highest concentration of each standard. Agilent 1200 series high performance auto sampler with an injection program was used to minimize carry-over effects.

#### 4.4.5. Recovery and Matrix Effect (or Ion Suppression)

Three different levels (Low: 0.1 mM, Medium: 1 mM and High: 10 mM, *n* = 3) of the seven analytes were added to the in vitro batch fermentation sample to assess recovery. After samples were processed according to Section 4.3 and analysed by the present technique, the recovery was calculated as (final calculated concentration-non spike concentration/added known concentration) × 100.

The recovery of known concentration (1 mM) in six different animal tissues, milk and plasma were assessed by spiking individual analytes and analysed by LC-MS/MS. The above formula was used to calculate the recovery in different matrices.

The matrix effect was calculated by the post-extraction spike method as indicated by RSC guidelines for LC-MS measurements [28]. The endogenous individual analytes are present in different matrices, therefore isotope labelled internal standards were used to assess matrix effects. The same concentrations (0.5 mM except lactate 0.25 mM for all tissues, milk and plasma, 5 mM acetate, 0.25 mM lactate and 0.5 mM others for in vitro batch fermentation sample) of individual compounds (isotope labelled internal standards) were spiked in different extracted matrices and acidified (0.5% ortho-phosphoric acid) The equation (Peak area in water-peak area in matrices/peak area in water) × 100 was used to assess the LC-MS/MS matrix effect for all analytes.

### 4.5. Data Analysis

Data files were exported and analysed on an Agilent MassHunter Quantitative analysis B.06.00/Build 6.0.388.0 (Agilent Technologies, Santa Clara, CA, USA) The software integrates the peak area for the metabolites which is then exported as an Excel document. The concentration of the SCFAs was calculated using the equation of the standard curve and the peak area ratio (analyte peak area/internal standard peak area) of the SCFA.

## 5. Conclusions

We have developed a new LC-MS technique for SCFAs analysis that involves less laborious sample preparative steps, does not require sample derivatisation and uses isotopically labelled internal standards to account for matrix effects which allows accurate measurement of SCFAs, BSCFAs and lactate in different biological matrices using LC-MS/MS. The application of this analytical tool to ex vivo and in vitro models will be instrumental in carrying out mechanistic studies to elucidate biological profiles and physiological effects of SCFAs and their related metabolites contributing to our overall understanding of the role of the microbiome in health and disease.

## Figures and Tables

**Table 1:** LC-MS/MS parameters of SCFA.

Analyte	Retention Time (mins)	Precursor Ion (*m/z*)	Product Ion (*m/z*)	Collision Energy	Cell Accelerator Energy	Polarity
Acetate	1.5 (2.3) *	61.1	43	16	4	Positive
D4-Acetate	1.5 (2.3) *	65.1	47	14	4	Positive
Butyrate	3.7 (4.9) *	89.1	43.1	14	4	Positive
13C2-Butyrate	3.7 (4.9)	91.1	44	14	4	Positive
Isobutyrate	2.9 (4.9) *	89.1	43.1	14	4	Positive
D6-Isobutyrate	2.9 (4.9)	95	49	14	4	Positive
Iso-Valerate	4.2 (5.4) *	103.1	43	14	4	Positive
D9-Isovalerate	4.2 (5.4) *	112.2	50.2	18	4	Positive
Lactate	1.7 (2.0) *	89	42.9	10	5	Negative
13C3-Lactate	1.7 (2.0) *	92	46	10	4	Negative
Propionate	2.3 (3.6) *	75	29	18	4	Positive
D2-Propionate	2.3 (3.6) *	77	31.1	14	4	Positive
Valerate	4.7 (5.4) *	103.1	75	10	4	Positive
D9-Valerate	4.7 (5.4) *	112.1	80	10	4	Positive

Note: * The figures in parentheses indicate the retention times obtained using a Phenomenex PFP column.

**Table 2:** Method performance data for individual SCFAs, BSCFAs and lactate in acidified water.

Analyte	R^2^	Precision (*n* = 6) (Intra-Day) R.S.D. %			Precision (*n* = 5) (Inter-Day) R.S.D. %			Accuracy (R.S.D. %)			LOD (mM)	LOQ (mM)
		L	M	H	L	M	H	L	M	H		
Acetate	0.998	11.3	3	2.9	19.3	4.1	6.1	98.2	103	96.6	0.003	0.009
Butyrate	0.999	4.6	2.4	3.6	16.7	5.4	4.5	120.4	102.1	99.8	0.001	0.003
Isobutyrate	0.999	2	1.7	2	10.2	7.4	2.3	107.8	107.9	102.6	0.001	0.003
Isovalerate	0.998	6	3.2	2.7	11.8	4.2	4.7	120	119.3	100.2	0.001	0.003
Lactate	0.999	2.6	2	1.7	9.5	7.6	2.2	120	104.9	98.6	0.001	0.003
Propionate	0.999	5.2	2	1.7	10	9	3.9	119.8	108.4	105.2	0.001	0.003
Valerate	0.998	8	4.4	3.9	14.7	8.4	5.3	116.3	111.4	92.9	0.001	0.003

**Table 3:** Recovery and matrix effect of spiked individual SCFAs, BSCFAs and lactate in appropriate matrices.

Sample Name	Acetate		Butyrate		Isobutyrate		Isovalerate		Lactate		Propionate		Valerate	
	Recovery (%)	Matrix Effect (%)	Recovery (%)	Matrix Effect (%)	Recovery (%)	Matrix Effect (%)	Recovery (%)	Matrix Effect (%)	Recovery (%)	Matrix Effect (%)	Recovery (%)	Matrix Effect (%)	Recovery (%)	Matrix Effect (%)
Brain	69	0	89	5	82	0	92	22	47	22	79	0	93	57
Faecal	107	0	112	50	103	0	115	46	102	24	100	0	121	56
Kidney	89	17	97	33	96	0	107	34	96	37	104	0	109	53
Liver	81	32	96	53	97	0	115	65	64	38	95	0	105	75
Milk	95	34	79	11	76	0	112	0	97	0	92	0	109	0
Muscle	71	0	89	4	94	0	107	31	75	52	91	0	108	59
Plasma	96	17	89	0	80	0	107	0	64	0	95	0	109	0
Spleen	95	14	98	76	93	0	111	80	95	38	99	0	100	84

Recovery was calculated as (final calculated concentration-non spike concentration/added known concentration) × 100.

**Table 4:** Recovery and matrix effect of spiked individual SCFAs, BSCFAs and lactate in spiked colon models.

Analytes Added	Measured Conc. (mM), Mean	Recovery (%)	CV (%)	Matrix Effect (%)
Acetate				
0	1.97	-	5.79	43.2
L (0.1 mM)	2.07	98	0.33	42.7
M (1.0 mM)	3.10	113	0.02	42.5
H (10 mM)	11.6	97	1.88	37.7
Butyrate				
0	0.13	-	-	0
L (0.1 mM)	0.25	120	3.85	0
M (1.0 mM)	1.21	108	0.01	0
H (10 mM)	10.1	100	0.61	0
Isobutyrate				
0	0.05	-	2.04	2.50
L (0.1 mM)	0.17	116	3.64	11.21
M (1.0 mM)	1.12	107	0.02	9.47
H (10 mM)	9.83	98	1.60	14.98
Isovalerate				
0	0.01	-	6.25	18.44
L (0.1 mM)	0.12	109	5.09	17.52
M (1.0 mM)	1.01	100	0.04	16.57
H (10 mM)	8.85	88	1.39	18.41
Lactate				
0	0.16	-	5.90	23.98
L (0.1 mM)	0.28	117	1.96	29.43
M (1.0 mM)	1.21	105	0.02	32.08
H (10 mM)	9.61	95	0.87	22.78
Propionate				
0	0.15	-	8.48	7.13
L (0.1 mM)	0.27	116	5.64	14.85
M (1.0 mM)	1.24	109	0.01	10.67
H (10 mM)	9.81	97	0.12	16.08
Valerate				
0	0.02	-	20.18	10.86
L (0.1 mM)	0.14	117	5.26	9.41
M (1.0 mM)	1.22	120	0.05	3.01
H (10 mM)	10.0	100	1.94	7.36

**Table 5:** A comparison of previous techniques with the new technique reported here.

Methodological Consideration	GC-MS	HPLC	LC-MS/MS	Current
Derivatization	Yes	No	Yes	No
Sensitivity	µM range	mM range	µM range	µM range
Instrument run time	Long	Long	Medium	short
Sample preparation	Laborious	Simple	Laborious	Simple
BSCFA detection	Yes	No	Yes	Yes
Lactate detection	No	Yes	No	Yes
Tested matrices	4	3	3	9
Sample preparation time	Long	Long	Long	Short (~10 min)
Instrument run time	14–45 min	45–75 min	14–35 min	10 min

## Data Availability

The data presented in this study are available on request from the corresponding author.

## Sample Availability

Standards from which the compounds were analysed are available from the stated commercial manufacturers. Biological samples are not available.
